# Pulmonary embolism after a single-stage, combined anterior and posterior approach lumbar surgery

**DOI:** 10.12669/pjms.296.3668

**Published:** 2013

**Affiliations:** Shujie Tang, MD, PhD, Department of Traditional Chinese Medicine, Medical school, Jinan University, Guangzhou, 510632, China.

**Keywords:** Pulmonary embolism, Lumbar burst fracture, Single-stage, Combined anterior and posterior approach

## Abstract

Pulmonary embolism is a fatal complication in orthopaedics surgery. While, the incidence of this life-threatening condition is low in spine surgery and few detailed reports have been published in English literatures. We present a case of pulmonary embolism which occurred after a single-stage, combined anterior and posterior approach surgery for L2 burst fracture. Although positive and timely rescue measures were performed, the patient died finally. We report the case to help spine surgeons to be aware of and take precautions against the fatal condition in spine surgery.

## INTRODUCTION

Pulmonary embolism is a lethal condition associated with orthopaedics surgery, particularly those with joint arthroplasty.^1^^-^^4^ In contrast, the incidence of the fatal complication resulted from spine surgery is low.^5^ In Pubmed, some authors focused on pulmonary embolism in spine surgery, but few detailed case reports are available.

In this manuscript, we present a case of pulmonary embolism which occurred after a single-stage, combined anterior and posterior approach surgery for L2 burst fracture, and the complication occurred seven days after surgery. Although positive rescue measures were performed, the patient died finally. The spine surgeons may neglect the prevention of the life-threatening complication because of its rarity, and fail in recognizing it immediately once it occurs. We report the case to help spine surgeons to be aware of and take precautions against the fatal condition.

## CASE REPORT

A 33-year-old male fell from eight meters height and complained of back pain when he was admitted to our hospital shortly after injury. Physical examination demonstrated stable vitals, but paraesthesia over the L1-2 distribution and decreased muscle force in both lower limbs. Deep tendon reflexes, perianal sensation and sphincter tone were normal. X-radiographs revealed loss of anterior body height, widening of the pedicles, anterior comminution and segmental kyphosis of L2 vertebral body. Magnetic resonance images revealed spinal canal narrowing at L2 level because of retropulsed bone fragments. The patient was diagnosed with L2 burst fracture.

A single-stage, combined anterior and posterior approach surgery was performed two days after injury. Corpectomy was performed on L2 vertebral body and a titanium cage filled with cancellous bone was placed between L1 and L3. Pedicle screw fixation system was used posteriorly to stabilize the titanium cage. The operation lasted 175 minutes and no complications occurred. The postoperative course remained uneventful, the patient was mobilized with crutches and orthosis two days later. In addition, the cutaneous sensation and muscle forces improved greatly, and no signs and symptoms associated with deep vein thrombosis were detected after surgery.

However, the patient suddenly lost consciousness seven days after surgery and had cardiopulmonary arrest. Cardiopulmonary resuscitation was performed immediately. The result of myocardial enzymes excluded the occurrence of acute myocardial infarction, but the D-dimers were found to be raised significantly. The patient was diagnosed as pulmonary embolism primarily and urokinase was injected via vein. However, the resuscitation was unsuccessful and the patient died at last. A post-mortem examination was performed and the result revealed the cause of death is pulmonary embolism.

## DISCUSSION

Pulmonary embolism is a fatal complication following spine surgery. Compared to joint arthroplasty, the incidence of pulmonary embolism is relatively low in spine surgery. In the clinical study of a total of 47,743 patients, Masuda reported the incidence of pulmonary embolism is 0.1%.^6^ In the analysis of 4,505,556 patients, Senders reported the incidence is 0.2%.^5^ However, the mortality of the complication is very high. In the current case, although diagnosis and rescue were performed immediately, the patient died finally. The complication should be paid more attention in spine surgery.

Pulmonary embolism results mostly from deep vein thrombosis which is related closely to injury of vessel wall, slowing of blood flow and blood hypercoagulation. The current case was performed a single-stage, combined anterior and posterior approach surgery. Compared to anterior or posterior approach, the combined approach presented with longer duration of anaesthesia, longer operation time and much blood loss in surgery.^7^ In a clinical study from Knob^8^, the mean operation time of anterior approach is 218 minutes, posterior approach is 134 minutes and the anterior-posterior approach is 254 minutes. The mean blood loss in anterior approach is 876 cc, posterior approach 828 cc, while anterior-posterior approach 1387 cc. The combined anterior and posterior approach differed from posterior and anterior approach in operation time and blood loss significantly, which was regarded as the risk factors for postoperative pulmonary embolism.^6^ In addition, some authors have suggested the combined anterior and posterior approach itself was the risk factor of postoperative pulmonary embolism.^5^^,^^9^ Compared to anterior or posterior approach, the single-stage, combined anterior and posterior approach result in more surgical trauma and longer bed rest period, which may affect adversely the hemodynamics of the patients, cause blood hypercoagulation, and lead to the formation of thrombosis and occurrence of pulmonary embolism.

Moreover, many cases of pulmonary embolism, including our case, presented with a sudden onset, which is related to the acute obstruction of one or more pulmonary arteries.^10^ The frequently observed symptoms of pulmonary embolism are dyspnea, tachycardia, and pleuritic chest pain, which are non-specific and usually appear in other cardiopulmonary diseases.^10^ Therefore, the early diagnosis and appropriate management are challenging**.**^11^ In many cases, the fatal condition may not be diagnosed correctly until the results of the autopsy are known. Subsequently, the prevention may be more important than its treatment.

Those patients undergoing spine surgery, especially single-stage, combined anterior and posterior approach surgery, should be monitored carefully for the development of deep vein thrombosis and pulmonary embolism. In terms of prevention, there is no clear consensus in thromboprophylaxis in spinal surgery.^12^ According to the guidelines of American College of Chest Physician, low molecular weight heparin(LMWH) was recommended as primary use in trauma patients.^13^ In addition, increasing the amount of ambulatory movement is regarded as effective measures for postoperative patients.^14^ LMWH can accelerate the antithrombin-mediated neutralization, thereby limiting thrombin generation.^15^ However, these prevention measures have been neglected by many spine surgeons.

This case report describes a rare complication of pulmonary embolism after a single-stage, combined anterior and posterior approach surgery. Familiarity with this complication will help spine surgeons in early diagnosis, therapy and prevention of pulmonary embolism, and decrease the mortality of the catastrophic phenomenon.
